# Alignment restoration of the hip and the influence on outcomes following DAA THA in none-obese and obese patients: a retrospective comparative study

**DOI:** 10.1186/s13018-026-07263-8

**Published:** 2026-10-16

**Authors:** Sebastian von Hertzberg-Boelch, Kilian List, Ioannis Stratos, Philip Marc Anderson, Axel Jakuscheit, Maximilian Rudert

**Affiliations:** Department of Orthopaedic Surgery, Julius-Maximilian University Wuerzburg, Wuerzburg, Germany

**Keywords:** Direct anterior approach, Total hip arthroplasty, Obesity, Restoration, Alignment

## Abstract

**Background:**

Accurate biomechanical alignment is crucial for the success of Total Hip Arthroplasty (THA). Obesity poses an increasing challenge in THA. This study compares hip restoration and clinical outcomes after direct anterior approach (DAA) THA between non-obese and obese patients.

**Methods:**

Biomechanical alignments after DAA THA were evaluated on postoperative radiographs by global, acetabular and femoral offsets as well as leg length and abductor lever arm. Restoration was calculated as the difference of the operated hip to the contralateral native joint (Δ). Precision was assessed using ± 5 mm and ± 2,5 mm target zones. Significant alignment parameters influencing clinical outcomes were evaluated using one-year postoperative WOMAC scores and compared between obese (BMI ≥ 30 kg/cm^2^) and non-obese (BMI < 30 kg/cm^2^) patients.

**Results:**

No significant differences in biomechanical restoration (*N* = 494) were found between obese and non-obese patients. Precision within the ± 5 mm target zones ranged from 31,4% to 73,5%, while precision within the ± 2,5 mm target zone ranged from 12,1% to 35,8%. Precision was independent of BMI. The abductor lever arm (ALA) alignment was the only radiographic parameter that correlated with clinical outcome (*N* = 344). In the non-obese, the one-year postoperative WOMAC score was significantly better when ΔALA was ≥ 0 mm (*p* = 0.023). However, this correlation was not observed in obese patients (*p* > 0.05). In obese patients, WOMAC scores were significantly better when ΔALA was ≥ 2 mm (*p* = 0.003).

**Conclusion:**

Accuracy of biomechanical restoration following DAA THA is comparable in non-obese and obese patients. To improve clinical outcomes, reduction of ΔALA should be avoided; obese patients may even benefit from a slight increase of ΔALA.

## Introduction

Total hip arthroplasty (THA) is a highly successful procedure. Modern implants demonstrate well-documented mid- to long-term outcomes, with implant survivor-ship rates exceeding 95% after ten years [1].

Accurate restoration of biomechanical alignment following THA is essential for joint function, achieving good clinical outcomes, reducing dislocation risk, and enhancing implant longevity [2, 3]. Different acetabular preparation techniques have been described: the conventional reaming technique, placing the cup at the true floor of the acetabulum, thereby medializing the center of rotation and reducing the horizontal acetabular offset (hAO) [2, 4]; and the anatomical reaming technique, reaming only to the subchondral bone to restore the anatomical center of rotation and hAO [2, 5]. Regardless of the positioning of the postoperative center of rotation, care should be taken to restore the global offset (GO) by adjusting the femoral offset (FO) to maintain hip abductor function [2, 6, 7, 5, 8]. However, in the past, increasing GO was often avoided due to concerns about patients complaints related to expanded muscle tension [9].

Minimally invasive approaches are increasingly used for THA to reduce recovery time and length of hospitalization [10–12]. Among these, the direct anterior approach (DAA) is the most anatomical minimally invasive approach, as it follows an intermuscular and internerval plane [13]. Consequently, the DAA has gained popularity in the last decade, accounting for up to 45% of all THA procedures in the US and 19,4% in Germany [10, 13]. However, there are concerns, that implant placement through minimally invasive approaches is less accurate compared to standard approaches [14].

Soft tissues management around the hip presents a specific challenge during minimally invasive THA, especially with the DAA [15, 16]. At the same time, registry data indicate that the average THA patient for THA is overweight [17], with obesity rates exceeding 25% [18]. There is ongoing debate about whether the DAA increases the risk for infection and wound complication in obese patients [19] and data on the suitability of the DAA for accurate hip joint restoration in this subgroup remain limited [20]. Even less is known about implant placement accuracy and its correlation with clinical outcome in obese patients.

Therefore this study aimed to: (I) compare the restoration of radiographic biomechanical alignment, between obese and none-obese patients undergoing DAA THA; (II) evaluate the precision of restoring hip anatomy relative to BMI, (III) analyze whether clinical outcome differs between obese and non-obese patients; and (IV) determine whether specific radiographic parameters of alignment influence clinical outcome one year after DAA THA.

## Methods

### Study design, THA implantation and patient inclusion

This single-center observational study was exempt from review board approval by the institutional committee. Patients were recruited from those who underwent cementless DAA THA between 2017 and 2018 under the care of one of eight senior surgeons (*N* = 688). At the study institution, the DAA has been the standard approach for THA since 2008. Each procedure was templated preoperatively using anteroposterior radiographs obtained in the supine position. The procedure is performed through the Hueter’s interval, with the patient in supine position and mobile leg draping, assisted by three surgical assistants. The detailed technique is described elsewhere [21]. An anatomical reaming technique is used to prepare the acetabulum for cup implantation, where reaming continues until reaching the subchondral bone. Additional reaming slightly medially and superiorly is performed as needed to achieve adequate press fit. The technique is adapted to the patient’s anatomy and at the surgeon’s discretion. A hemispherical but flattened cup with a rough-blasted surface (Allofit Alloclassic S, ZimmerBiomed, Germany) is impacted using offset instruments. For the femur, a proximally plasma-sprayed monoblock stem with cementless metaphyseal wedge fixation is used (ML Taper, ZimmerBiomed, Germany). The stem is available with standard and lateralized neck option. Intraoperative fluoroscopy was used to confirm the final implant position after implantation.

Patients were excluded if they received non-standard implants (*N* = 22), had radiographs of insufficient quality (*N* = 7), had contralateral THA (*N* = 159), or had missing BMI data (*N* = 6). This left 494 patients for postoperative radiographic analysis. Their demographic data are presented in Table 1. According to the World Health Organization patients with BMI ≥ 30 kg/cm^2^ were considered as obese [22].

**Table 1:** Demographic data for non- obese and obese patients

	Non obese (N=336)	Obese (N=158)	*p*
BMI (kg/m^2^) Mean and SD	25,8 (2,7)	34,6 (4,6)	<0.001
Women N (%)	183 (54,5)	84 (53,2)	0.787
Age mean and SD	64,0 (11,6)	63,9 (10,3)	0.918
Surgery time (min) mean and SD	58,9 (17,0)	65,3 (19,0)	<0.001
Cup size mean and SD	52 (3,1)	52.6 (3,0)	0.059
Stem size median and range	11 (4–17,5)	11 (6–17,5)	0.86
Length of stay (days) median and range	6 (2–23)	6 (3–27)	0.016
Complications N (%) Wound healing and infection Fracture Dislocation Cup Loosening	11 (3,3) 2 4 4 1	10 (6,3) 6 2 1 1	0.116

### Radiographic assessment

**Fig. 1:**
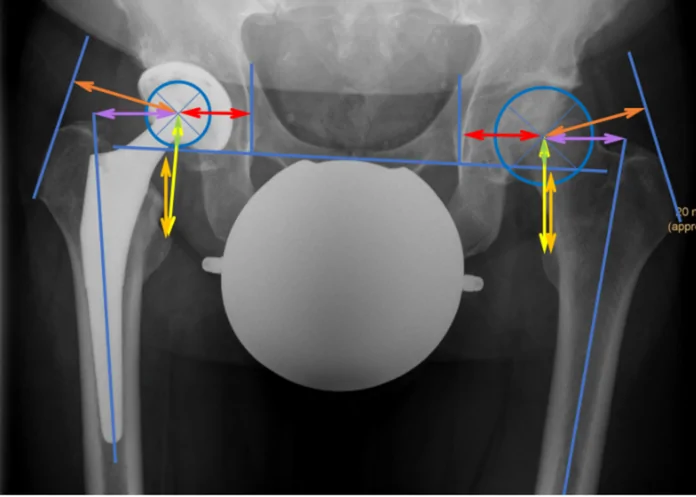
Postoperative radiograph depicting measurement technique of biomechanical parameters of the operated and contralateral native hip; red line hAO; green line vAO; purple line hFO; yellow line vFO; orange line LL, brown line ALA

The mediCAD THA planning software (medicad Hectec, Germany) was used to measure the biomechanical alignment parameters on postoperative anteroposterior (ap) radiographs (supine ap pelvic radiograph with the center beam on the symphysis and approximately 20° of internal rotation of both thighs). A radiograph was considered inadequate for radiographic assessment if the longitudinal axis of the coccyx was displaced by more than 1 cm from the symphysis pubis and the obturator foramina were asymmetric. The measured parameters were:

- Horizontal Acetabular Offset (hAO): The distance from the center of the femoral head to a line drawn at the acetabular wall perpendicular to the inter-teardrop line [17] (red line in Fig. 1).
- Vertical Acetabular Offset (vAO): The distance from the center of the femoral head to the inter-teardrop line [17] (green line in Fig. 1).
- Horizontal Femoral Offset (hFO): The shortest distance from the femoral shaft axis to the center of the femoral head [17] (purple line in Fig. 1).
- Vertical Femoral Offset (vFO): The distance from the lesser trochanter to the center of the femoral head [8] (yellow line in Fig. 1).
- Leg Length (LL): The distance from the inter-teardrop line to a perpendicular line drawn to the lesser trochanter [17] (orange Line in Fig. 1).
- Abductor Lever Arm (ALA): The distance from the center of the femoral head to the tangent of the greater trochanter [9] (brown line in Fig. 1).

Global Offset (GO) was calculated as the sum of hAO and hFO [8]. Additionally, anteversion and Cup inclination were measured and calculated as described by Lewinnek et al. [23].

All measurements were performed by two orthopaedic research fellows. For each single parameter the difference from operated side to the contralateral side was calculated (Δ).

To quantify precision of biomechanical restoration, target zones were used. The limits of the target zones were adopted from previous studies:

- Within ± 5 mm compared to the contralateral side [6–9].
- Within ± 2,5 mm [8].

Although target zones for FO and AO have not been explicitly described in the literature, the aforementioned limits were applied for these parameters. Precision was assessed as the percentage of cases within the predefined target zones of ± 5 mm and ± 2.5 mm, stratified by BMI group.

### Outcome assessment

The WOMAC Score was prospectively assessed preoperatively and one year postoperatively. The WOMAC Score is joint-specific and evaluates three dimensions of complaints (Pain (5 questions), stiffness (2 questions), and physical function (17 questions)) from no symptoms to extreme symptoms. For analysis, ratings were converted to the Likert version of the WOMAC score, which uses an ordinal scale from 0 to 4 for each question [24]. The maximum score of the three dimensions are 20 points for pain, 8 points for stiffness and 68 for physical function. The overall index score is the sum of these three dimensions. Lower scores indicate fewer complaints. The Minimal Clinically Important Improvement after THA is reported to be between 7 and 10 score points [25].

For outcome analysis, those patients with complications (*N* = 21) and those with missing WOMAC score (*N* = 129) were excluded, leaving 344 patients. The preoperative measurements are shown in Table 2.

**Table 2:** Preoperative WOMAC score for non-obese and obese patients; p< 0.05 was significant

	Non obese (N=247)	Obese (N=97)	*p*
WOMAC index	50,8 (17,7)	56,9 (17,2)	*0.004*
WOMAC pain	10,5 (3,7)	11,5 (3,8)	*0.029*
WOMAC stiffness	4,3 (2,1)	4,7 (1,9)	0.088
WOMAC physical function	36,0 (13,3)	40,7 (12,9)	*0.003*

To quantify the benefit, the residual complaints were calculated as proportion of the postoperative WOMAC score relative to the preoperative WOMAC score.

### Data analyses

Numeric data is shown as mean and standard deviation and groups were compared with the student’s’ t test after testing for equality of variance with the Leven test. Ordinal data is shown as median and range and was compared with the Man-Whitney-U-Test. Correlations were evaluated with the *Pearson* -test for numeric data and with *Spearman* -test for not numeric data. Contingency of variance were assessed with *Pearson Chi square* -test. *P* was set at < 0.05.

## Results

### Biomechanical alignment restoration of the hip joint (I)

Biomechanical alignment restoration of the hip joint showed no significant differences between obese and the non-obese patients as summarized in Table 3. For instance, ΔhAO values were − 3,4 ± 4,5 mm in non-obese patients and − 3,5 ± 4,2 mm in obese patients (*p* = 0,933), while ΔhFO values were 2,7 ± 6,5 mm and 2,5 ± 7,4 mm, respectively (*p* = 0.768).

**Table 3:** Comparison of radiographic parameters between obese and non-obese patients

	Inclination (degrees)	Anteversion (degrees)	Δ GO (mm)	Δ hAO (mm)	Δ vAO (mm)	Δ hFO (mm)	Δ vFO (mm)	Δ ALA (mm)	Δ LL (mm)
non-obese	36,7 (7,2)	25,9 (4.9)	−0,8	−3,4 (4,5)	4,6 (4,1)	2,7 (6,5)	7,4 (4,7)	−0,9 (6,1)	2,6 (5,9)
obese	37,3 (6,1)	25,0 (5.0)	−1,0	−3,5 (4,2)	5,0 (3,9)	2,5 (7,4)	8,2 (5,7)	−1,7 (6,4)	3,3 (6,1)
*p*	0.393	0.550	0.735	0.933	0.379	0.768	0.104	0.237	0.204

hAO: horizontal Acetabular Offset; vAO vertical Acetabular Offset; hFO: horizontal Femoral Offset; vFO: vertical Femoral Offset; ALA: Abductor Lever Arm; LL Leg length; Δ: Difference operated − nativ side (i.e. Δ GO was medialized in comparison to the contralateral hip)

Precision (II), defined by restoration in the ± 5 mm and ± 2,5 mm target zones, did not correlate with BMI (Table 4). For instance, 58,1% of all patients achieved restoration within the ± 5 mm target zone for GO, while only 26,1% met the stricter ± 2,5 mm target zone. The correlation across BMI categories was not significant, neither for the ± 5 mm target zone (*p* = 0.404) nor for the ± target zone (*p* = 0.607)

**Table 4:** Percentage for restoration in target zones ± 5 mm and ± 2,5 mm for radiographic parameters with *p* values of Pearson chi square according to BMI

Zone	± 5 mm						± 2.5 mm					
BMI	All (*N* = 494)	<=25 (*N* = 126)	25 to < = 30 (*N* = 210)	30 to < = 35 (*N* = 103)	> 35 (*N* = 55)	*p*	All (*N* = 494)	<=25 (*N* = 126)	25 to < = 30 (*N* = 210)	30 to < = 35 (*N* = 103)	> 35 (*N* = 55)	*p*
GO	58,1	57,1	56,7	65,0	52,7	0.404	26,1	26,2	27,1	26,2	21,8	0.607
ALA	58,3	67,5	56,2	51,5	58,2	0.081	30,4	36,5	29,0	28,2	25,5	0.350
vAO	59,1	57,1	60,5	59,2	58,2	0.944	26,1	26,2	30,0	21,4	20,0	0.271
hAO	73,5	68,3	74,3	75,8	80,0	0.373	35,8	38,9	37,1	31,1	32,7	0.590
vFO	31,4	31,7	33,3	35,0	16,4	0.080	12,1	12,7	13,8	13,6	1,8	0.099
hFO	57,9	56,3	60,5	53,4	60,0	0.971	29,8	28,6	32,4	21,4	38,2	0.825
LL	60,7	63,5	59,5	60,2	60,0	0.906	31,2	28,6	31,9	28,2	40,0	0.410

hAO: horizontal Acetabular Offset; vAO vertical Acetabular Offset; hFO: horizontal Femoral Offset; hVO: vertical Femoral Offset; ALA: Abductor Lever Arm; LL Leg length;

### Outcome (III)

There was no significant difference in outcome between the non-obese and obese patients (Table 5). The mean difference was below the threshold for Minimal Clinically Important Improvements.

**Table 5:** Comparison of outcome between obese and non obese patients

	WOMAC post (points)	Residual complaints (%)	WOMAC pain (points)	WOMAC stiffness	WOMAC function
Obese N=97 mean and SD	17,2 (15,7)	33,4 (38,7)	2,6 (3,1)	1,7 (1,5)	12,8 (11,8)
Non-obese N=247 mean and SD	15,3 (16,7)	30,5 (31,8)	2,6 (3,4)	1,5 (1,6)	11,3 (12,4)
*p*	0.353	0.474	0.838	0.304	0.291

### Influence alignment restoration on outcome (IV)

**Fig. 2:**
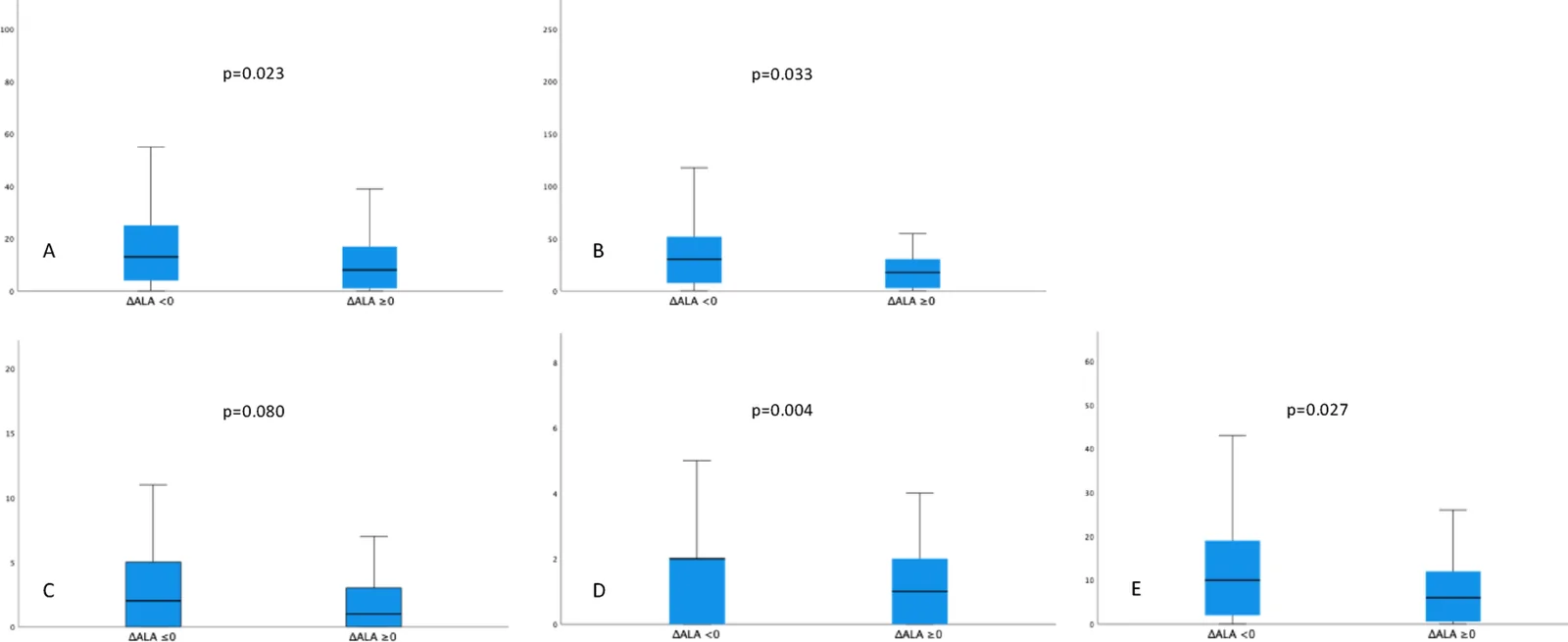
Boxplots comparing WOMAC scores of non-obese patients at a ΔALA threshold of 0 mm. **A** WOMAC Index; **B** Residual complaints; **C** WOAMC pain; **D** WOMAC stiffness; **E** WOMAC Function

Restoration of the radiographic parameters within the ± 5 mm and ± 2,5 mm target zones showed only isolated significant correlations with outcomes (Tables 6 and 7 in the appendix). Regardless of the target zones, ΔALA had the most correlations with outcomes in both groups (Tables 6 and 7 in the appendix). Larger ΔALA correlated with better outcomes. In the non-obese group, hips with a ΔALA of 0 mm or greater had significant better outcomes (Fig. 2; A–E). The mean differences for postoperative WOMAC, residual complaints, the subscore for pain, the subscore for stiffness and the subscore for function were 4.94, 8.79, 3.57, 0.76 and 0.61, respectively.

**Fig. 3:**
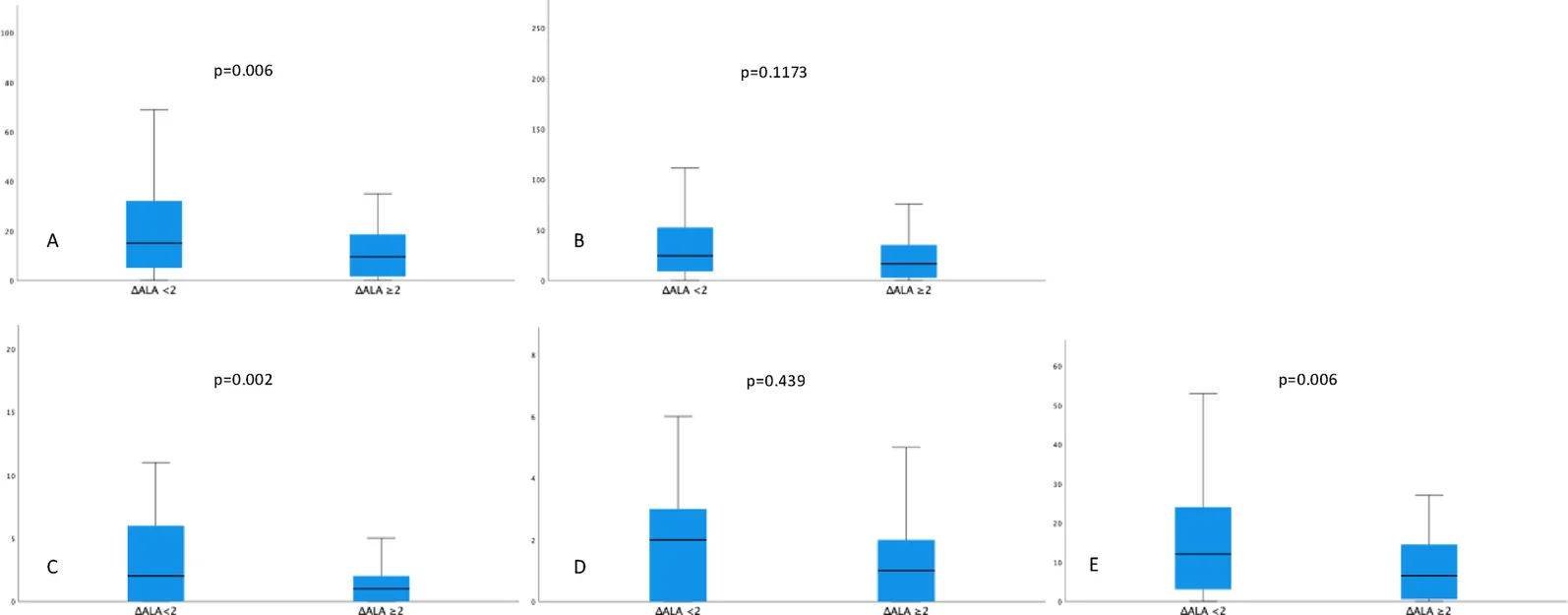
Boxplots comparing WOMAC scores of obese patients at a ΔALA threshold of 2 mm. **A** WOMAC Index; **B** Residual complaints; **C** WOMAC pain; **D** WOMAC stiffness; **E** WOMAC Function

In the obese-group, the same threshold value for ΔALA of 0 mm showed no significant differences in outcomes (*p* > 0.115). However, with a threshold for ΔALA of 2 mm or greater, the postoperative WOMAC score and the subscore for pain and the subscore for function were significantly better (Fig. 3A–E). The mean differences for the postoperative WOMAC score, the subscore for pain and the subscore for function were 8.14, 1.68 and 6.18, respectively.

## Discussion

This study demonstrates that obesity does not compromise implant placement accuracy in patients undergoing DAA THA when compared with non-obese patients. In both groups, overall precision of implant positioning was only moderate, as assessed using ± 5 and ± 2.5 mm target zones. Among all radiographic parameters, ALA was the only factor that showed a significant correlation with clinical outcome. At one year postoperatively, non-obese patients achieved significantly better WOMAC scores when ΔALA was not reduced, whereas obese patients tended to benefit from a slight increase in ΔALA.

Radiographic analysis revealed no significant differences in alignment restoration between obese and non-obese patients. However, surgery time was significantly longer in the obese group, reflecting increased technical difficulty. Despite this, implant placement accuracy was comparable between the two groups, indicating that longer surgical duration allowed surgeons to compensate for the increased complexity in obese patients.

Overall, only moderate precision was achieved regardless of BMI. This finding is consistent with previous studies investigating other approaches for THA [8, 26, 27, 28]. For example, the restoration of GO within the ± 5 mm target zone was achieved in only 58% of cases in our study, which aligns with results reported by Vorimore et al. in their large cohort of 500 THAs performed using an anterolateral or a posterior approach [8].

These findings suggest that conventional THA implantation techniques generally lack high precision, independent of BMI or surgical approach.

The relationship between radiographic hip reconstruction and clinical outcome remains incompletely understood. Kelly et al. categorized GO as increased (> 5 mm), matched, or decreased (< 5 mm ) in 499 patients undergoing DAA THA and found that decreased offset was associated with inferior outcome [5]. Similarly, Bullen et al. reported worse clinical results in patients with a hip offset reduction of 20 mm or more in a multicenter cohort of 414 [29]. Vorimore et al. observed significantly better outcomes in the patients whose GO and LL were restored within the ± 2,5 mm target zone; however, only 10% of their cohort achieved this level of precision [8]. The authors noted an average medialization of AO by 2 mm but did not differentiate between vertical and horizontal changes.

In contrast, our analysis (Table 3) demonstrated both medialization and (indicated by hAO) proximalization (indicated by vAO) of the hip center of rotation. These changes were compensated by corresponding alterations in femoral offset (hFO and vFO). The ALA - unlike GO - captures both, vertical and horizontal offset changes mor effectively, making it a more appropriate radiographic surrogate for the abductor muscle function [9]. Consistent with this concept, patients with a shortened ALA compared with the contralateral hip experienced significantly worse outcomes. In non-obese patients, maintaining ΔALA was critical, while obese patients appeared to benefit from a slight increase in ΔALA.

Concerns that increasing ALA might lead to excessive muscle tension were not supported by the findings of Bjordal et al. [9]. Furthermore, Trevisan et al. identified ALA as the only biomechanical parameter predictive of clinical outcome in their cohort of 95 patients undergoing DAA THA showing a positive correlation between increased ALA and improved outcomes [30]. Our results support these observations.

This study has several limitations. Its retrospective design and short-term follow-up preclude assessment of long term implant survival ant outcome. Moreover, 32,2% of patients included in the radiographic analysis did not complete the preoperative or postoperative WOMAC questionnaires. However, similar studies have reported non- responder rates exceeding 40% [8]. The comparison of clinical outcomes between obese and non-obese patients may also be underpowered, although Bullen et al. demonstrated significant correlations between WOMAC scores and hip reconstruction parameters in as subgroup of only 20 patients [29].

The most important limitation is that three-dimensional hip reconstruction was assessed using coronal plane radiographs only. Sagittal plane parameters, such as anterior femoral offset, were not evaluated. Depending on the radiographic parameter assessed, the interobserver reliability has been reported as good to excellent [8]. However, advanced imaging modalities, such as computed tomography, would be required for a comprehensive three-dimensional analysis [31].

The main strength of this study lies in the homogeneity of the study population. All patients were treated using the same THA protocol and implants by high-volume DAA surgeons, allowing for a focused evaluation of implant positioning and its clinical relevance.

## Conclusion

Implant placement precision in DAA THA is comparable between non-obese and obese patients. To improve clinical outcomes, reduction of the abductor lever arm should be avoided; in obese patients, a slight over-restoration of the abductor lever arm may be beneficial.

## Data Availability

The datasets used and analysed during the current study are available from the corresponding author on reasonable request.

## Appendix

See Tables 6 and 7.

**Table 6:** Correlation coefficients and *p* (in brackets) between postoperative WOMAC score and alignment parameters as well as between postoperative WoMAC score and precision for obese patients; *p*> 0.05 was signficant

	GO			ALA			vAO			hAO		
	All	5	2.5	All	5	2.5	All	5	2.5	All	5	2.5
WOMAC index	− 0,144 (0.159)	0,014 (0.358)	0,012 (0.909)	**− 0,289 (0.012)**	− 0,024 (0.813)	0,034 (0.738)	− 0,044 (0.666)	− 0,114 (0.268)	0,169 (0.099)	0,122 (0.232)	0,094 (0.358)	0,046 (0.658)
Residual complaints	− 0,064 (0.530)	0,077 (0.454)	0,012 (0.906)	− 0,218 (0.060)	− 0,019 (0.853)	0,033 (0.750)	− 0,065 (0.530)	− 0,073 (0.475)	0,170 (0.095)	0,127 (0.217)	0,128 (0.211)	0,072 (0.486)
WOMAC pain	− 0,084 (0.411)	0,027 (0.792)	− 0,049 (0.635)	**− 0,308 (0.007)**	− 0,071 (0.488)	0,015 (0.883)	− 0,097 (0.347)	− 0,007 (0.946)	0,190 (0.062)	− 0,184 (0.071)	0,135 (0.187)	0,126 (0.219)
WOMAC stiffness	− 0,092 (0.370)	0,046 (0.654)	0,026 (0.801)	− 0,110 (0.346)	− 0,080 (0.436)	− 0,020 (0.843)	− 0,031 (0.766)	− 0,066 (0.521)	0,047 (0.647)	0,079 (0.442)	0,030 (0.771)	− 0,084 (0.415)
WOMAC function	− 0,157 (0.124)	0,014 (0.895)	0,022 (0.834)	**− 0,288 (0.012)**	− 0,015 (0.887)	0,055 (0.595)	− 0,030 (0.771)	− 0,138 (0.177)	0,169 (0.098)	0,105 (0.308)	0,070 (0.493)	0,029 (0.782)

	vFO			hFO			LL		
	All	5	2.5	All	5	2.5	All	5	2.5
WOMAC index	− 0,019 (0.855)	− 0,004 (0.966)	0,185 (0.070)	**− 0,216 (0.034)**	0,013 (0.897)	− 0,165 (0,107)	0,016 (0.879)	0,109 (0.290	0,116 (0.258)
Residual complaints	− 0,113 (0.269)	0,018 (0.862)	**0,203 (0.046)**	− 0,142 (0.164)	0,009 (0.931)	**− 0,201 (0,047)**	− 0,076 (0.457)	0,146 0.153)	0,170 (0.097)
WOMAC pain	− 0,054 (0.599)	0,059 (0.563)	0,150 (0.142)	− 0,198 (0.052)	0,006 (0.955)	− 0,136 (0.185)	0,021 (0.842)	0,094 (0.361)	0,165 (0.108)
WOMAC stiffness	− 0,024 (0.819)	0,005 (0.962)	0,065 (0.527)	− 0,138 (0.176)	0,006 (0.953)	− 0,126 (0.217)	− 0,015 (0,881)	0,145 (0.155)	0,082 (0.422)
WOMAC function	− 0,008 (0.939)	− 0,018 (0.858)	0,185 (0.070)	**− 0,217 (0.033)**	0,015 (0.885)	− 0,155 (0.129)	0,017 (0.865)	0,091 (0.377)	0,093 (0.363)

hAO: horizontal Acetabular Offset; vAO vertical Acetabular Offset; hFO: horizontal Femoral Offset; hVO: vertical Femoral Offset;ALA: Abductor Lever Arm; LL Leg length

**Table 7:** Correlation coefficients and *p* (in brackets) between postoperative WOMAC score and alignment parameters as well as between postoperative WoMAC score and precision for obese patients; *p*> 0.05 was signficant

	GO			ALA			vAO			hAO		
	All	5	2.5	All	5	2.5	All	5	2.5	All	5	2.5
WOMAC index	− 0,072 (0.260)	0,010 (0.882)	0,038 (0.552)	**− 0,177 (0.010)**	− 0,114 (0.072)	− 0,105 (0.101)	− 0,017 (0.793)	0,097 (0.130)	− 0,053 (0.403)	− 0,025 (0.700)	0,011 (0.867)	− 0,042 (0.507)
Residual complaints	− 0,084 (0.187)	− 0,046 (0.476)	− 0,038 (0.553)	**− 0,167 (0.015)**	− 0,093 (0.147)	− 0,079 (0.218)	0,003 (0.957)	0,094 (0.139)	− 0,046 (0.476)	0,011 (0.864)	0,031 (0.633)	− 0,038 (0.553)
WOMAC stiffness	− 0,095 (0.138)	0,079 (0.219)	0,069 (0.277)	**− 0,213 (0.002)**	**− 0,140 (0.028)**	− 0,042 (0.506)	− 0,057 (0.373)	0,105 (0.101)	− 0,020 (0.760)	− 0,003 (0.968)	0,036 (0.572)	0,016 (0.797)
WOMAC pain	− 0,037 (0.562)	0,058 (0.365)	0,056 (0.378)	**− 0,168 (0.014)**	− 0,096 (0.131)	− 0,112 (0.080)	− 0,020 (0.750)	**0,131 (0.040)**	− 0,011 (0.868)	0,013 (0.837)	0,055 (0.389)	− 0,012 (0.847)
WOMAC function	− 0,074 (0.244)	− 0,013 (0.835)	0,021 (0.740)	**− 0,165 (0.016)**	− 0,099 (0.119)	− 0,068 (0.285)	− 0,010 (0.880)	0,089 (0.163)	− 0,068 (0.285)	− 0,036 (0.570)	− 0,004 (0.947)	− 0,052 (0.417)

	vFO			hFO			LL		
	All	5	2.5	All	5	2.5	All	5	2.5
WOMAC index	− 0,046 (0.471)	0,061 (0.338)	0,032 (0.618)	− 0,058 (0.364)	0,051 (0.424)	0,001 (0.990)	− 0,027 (0.672)	− 0,099 (0.122)	− 0,015 (0.815)
Residual complaints	− 0,074 (0.250)	0,068 (0.287)	0,030 (0.636)	− 0,096 (0.134)	0,059 (0.356)	0,039 (0.540)	− 0,067 (0.297)	− 0,098 (0.123)	− 0,030 (0.644)
WOMAC stiffness	0,003 (0.967)	0,008 (0.900)	0,062 (0.336)	− 0,097 (0.128)	0,118 (0.064)	0,080 (0.211)	0,034 (0.595)	− 0,072 (0.261)	0,024 (0.711)
WOMAC pain	− 0,042 (0.516)	0,033 (0.605)	− 0,013 (0.844)	− 0,048 (0.516)	0,053 (0.408)	0,059 (0.356)	− 0,032 (0.617)	− 0,053 (0.407)	0,002 (0.975)
WOMAC function	− 0,051 (0.423)	0,069 (0.281)	0,032 (0.619)	− 0,052 (0.411)	0,039 (0.539)	− 0,024 (0.710)	− 0,032 (0.615)	− 0,113 (0.075)	− 0,022 (0.729)

hAO: horizontal Acetabular Offset; vAO vertical Acetabular Offset; hFO: horizontal Femoral Offset; hVO: vertical Femoral Offset;ALA: Abductor Lever Arm; LL Leg length

## References

[1] Callanan MC, Jarrett B, Bragdon CR, Zurakowski D, Rubash HE, Freiberg AA, et al. The John Charnley Award: risk factors for cup malpositioning: quality improvement through a joint registry at a tertiary hospital. Clin Orthop Relat Res. 2011. 10.1007/s11999-010-1487-1. PMC3018230 20717858

[2] Kiyama T, Naito M, Shinoda T, Maeyama A. Hip abductor strengths after total hip arthroplasty via the lateral and posterolateral approaches. J Arthroplasty. 2010. 10.1016/j.arth.2008.11.001. 19359133

[3] Mahmood SS, Mukka SS, Crnalic S, Wretenberg P, Sayed-Noor AS. Association between changes in global femoral offset after total hip arthroplasty and function, quality of life, and abductor muscle strength. A prospective cohort study of 222 patients. Acta Orthop. 2016. 10.3109/17453674.2015.1091955. PMC4940589 26471772

[4] Bonnin MP, Archbold PH, Basiglini L, Fessy MH, Beverland DE. Do we medialise the hip centre of rotation in total hip arthroplasty? Influence of acetabular offset and surgical technique. Hip Int. 2012. 10.5301/HIP.2012.9350. 22865253

[5] Kelly P, Grant C, Cochrane N, Horn M, Florance J, Bolognesi M, et al. Decreasing Hip offset in total hip arthroplasty results in decreased physical function scores. J Arthroplasty. 2024. 10.1016/j.arth.2024.06.004. 38851407

[6] Renkawitz T, Weber T, Dullien S, Woerner M, Dendorfer S, Grifka J, et al. Leg length and offset differences above 5 mm after total hip arthroplasty are associated with altered gait kinematics. Gait Posture. 2016. 10.1016/j.gaitpost.2016.07.011. 27450670

[7] Luca DiGiovanni P, Gasparutto X, Armand S, Hannouche D. The modern state of femoral, acetabular, and global offsets in total hip arthroplasty: a narrative review. EFORT Open Rev. 2023. 10.1530/EOR-22-0039. PMC10026057 36916758

[8] Vorimore C, Innmann M, Mavromatis S, Speirs A, Verhaegen JCF, Merle C, et al. Impact of offset and leg length on functional outcomes post-total hip arthroplasty: how accurate should coronal reconstruction be? J Arthroplasty. 2024. 10.1016/j.arth.2024.06.017. 38897260

[9] Bjordal F, Bjorgul K. The role of femoral offset and abductor lever arm in total hip arthroplasty. J Orthop Traumatol. 2015. 10.1007/s10195-015-0358-7. PMC4633429 26068583

[10] Stratos I, Heller KD, Rudert M. German surgeons’ technical preferences for performing total hip arthroplasties: a survey from the National Endoprosthesis Society. Int Orthop. 2021. 10.1007/s00264-021-05188-x. PMC8930942 34904195

[11] Migliorini F, Pintore A, Eschweiler J, Oliva F, Hildebrand F, Maffulli N. Factors influencing the outcomes of minimally invasive total hip arthroplasty: a systematic review. J Orthop Surg Res. 2022. 10.1186/s13018-022-03168-4. PMC9118783 35585545

[12] Barrett WP, Turner SE, Leopold JP. Prospective randomized study of direct anterior vs postero-lateral approach for total hip arthroplasty. J Arthroplasty. 2013. 10.1016/j.arth.2013.01.034. 23523485

[13] Driesman A, Yang CC. Clinical outcomes of DAA and related techniques in hip arthroplasty. Arthroplasty. 2023. 10.1186/s42836-023-00198-z. PMC10472647 37653546

[14] Innmann MM, Streit MR, Kolb J, Heiland J, Parsch D, Aldinger PR, et al. Influence of surgical approach on component positioning in primary total hip arthroplasty. BMC Musculoskelet Disord. 2015. 10.1186/s12891-015-0623-1. PMC4523950 26242180

[15] Holzapfel BM, Rak D, Kreuzer S, Arnholdt J, Thaler M, Rudert M. Short stem hip arthroplasty via the minimally invasive direct anterior approach. OOT. 2021. 10.1007/s00064-021-00723-w. 34251469

[16] Corten K, Holzapfel BM. Direct anterior approach for total hip arthroplasty using the bikini incision. OOT. 2021. 10.1007/s00064-021-00721-y. 34338830

[17] Patient characteristics for primary hip replacement procedures [Internet]. 2023. Available from: reports.njrcentre.org.uk.

[18] Annual. Report 2023 EPRD Deutsche Endoprothesenregister gGmbH.

[19] Shah NVHH, Wolff DT, Newman JM, Pivec R, Naziri Q, Shah VR, Maheshwari AV. Does surgical approach for total hip arthroplasty impact infection risk in the obese patient? a systematic review. Orthopedics. 2022. 10.3928/01477447-20211227-03. 34978509

[20] Weissenberger M, Heinz T, Rak D, Stratos I, Anderson PM, Ludemann M, et al. Does Body mass index (BMI) affect the reconstruction of biomechanical parameters in patients undergoing total hip replacement (THR) through the direct anterior approach (DAA)? J Clin Med. 2024. 10.3390/jcm13020467. PMC10816045 38256601

[21] Rudert M. Zementfreie Hüftendoprothese: minimalinvasiver anteriorer Zugang. Berlin Heidelberg: Springer; 2019.

[22] Obesity WHO. Preventing and managing the global epidemic: report of a WHO consultation. Geneva: World Health Organization; 2000. 11234459

[23] Lu M, Zhou YX, Du H, Zhang J, Liu J. Reliability and validity of measuring acetabular component orientation by plain anteroposterior radiographs. Clin Orthop Relat Res. 2013. 10.1007/s11999-013-3021-8. PMC3734435 23645336

[24] Bellamy N. The WOMAC knee and hip osteoarthritis indices: development, validation, globalization and influence on the development of the AUSCAN hand osteoarthritis Indices. Clin Exp Rheumatol; 2005. 16273799

[25] Marques CJ, Pinnschmidt HO, Bohlen K, Lorenz J, Lampe F. TKA patients experience less improvement than THA patients at 3 and 12 months after surgery. A retrospective observational cohort study. J Orthop. 2020. 10.1016/j.jor.2020.09.007. PMC7511647 33013084

[26] Innmann MM, Maier MW, Streit MR, Grammatopoulos G, Bruckner T, Gotterbarm T, et al. Additive influence of hip offset and leg length reconstruction on postoperative improvement in clinical outcome after total hip arthroplasty. J Arthroplasty. 2018. 10.1016/j.arth.2017.08.007. 28887022

[27] Kayani BKS, Thakrar RR, Huq SS, Haddad FS. Assuring the long-term total joint arthroplasty: a triad of variables. Bone Joint J. 2019;1. 10.1302/0301-620X.101B1.BJJ-2018-0377.R1. 30648491

[28] Antoniadis A, Dimitriou D, Flury A, Wiedmer G, Hasler J, Helmy N. Is direct anterior approach a credible option for severely obese patients undergoing total hip arthroplasty? a matched-control, retrospective, clinical study. J Arthroplasty. 2018. 10.1016/j.arth.2018.03.071. 29729934

[29] Bullen ME, Babazadeh S, van Bavel D, McKenzie DP, Dowsey MM, Choong PF. Reduction in offset is associated with worse functional outcomes following total hip arthroplasty. J Arthroplasty. 2023. 10.1016/j.arth.2022.09.001. 36096271

[30] Trevisan C, Klumpp R, Piscitello S, Compagnoni R, Grattieri R, Cazzaniga C. Biomechanical reconstruction parameters obtained after direct anterior approach total hip arthroplasty do not compromise clinical outcome. Eur J Orthop Surg Traumatol. 2020. 10.1007/s00590-020-02727-x. 32613469

[31] Hirano Y, Imai N, Nozaki A, Horigome Y, Suzuki H, Kawashima H. The association of postoperative global femoral offset with total hip arthroplasty outcomes. Sci Rep. 2023. 10.1038/s41598-023-28863-y. PMC9884219 36709404

